# Identification, Characterization and Expression Analysis of Anthocyanin Biosynthesis-related *bHLH* Genes in Blueberry (*Vaccinium corymbosum* L.)

**DOI:** 10.3390/ijms222413274

**Published:** 2021-12-10

**Authors:** Yongyan Zhang, Fan Liu, Bin Wang, Huan Wu, Junwei Wu, Jiapeng Liu, Yueting Sun, Chunzhen Cheng, Dongliang Qiu

**Affiliations:** 1 College of Horticulture, Fujian Agriculture and Forestry University, Fuzhou 350002, China; zhyy0425@126.com (Y.Z.); lfan1111@126.com (F.L.); wb971220@163.com (B.W.); WUhuan980422@163.com (H.W.); a839941883@126.com (J.W.); a15555767583@163.com (J.L.); yuetingsun@126.com (Y.S.); 2 College of Horticulture, Shanxi Agricultural University, Jinzhong 030801, China

**Keywords:** basic helix-loop-helix protein (bHLH), blueberry, anthocyanin, gene expression, expression regulation

## Abstract

Basic helix-loop-helix proteins (bHLHs) play very important roles in the anthocyanin biosynthesis of many plant species. However, the reports on blueberry anthocyanin biosynthesis-related bHLHs were very limited. In this study, six anthocyanin biosynthesis-related bHLHs were identified from blueberry genome data through homologous protein sequence alignment. Among these blueberry bHLHs, VcAN1, VcbHLH42-1, VcbHLH42-2 and VcbHLH42-3 were clustered into one group, while VcbHLH1-1 and VcbHLH1-2 were clustered into the other group. All these bHLHs were of the bHLH-MYC_N domain, had DNA binding sites and reported conserved amino acids in the bHLH domain, indicating that they were all G-box binding proteins. Protein subcellular location prediction result revealed that all these bHLHs were nucleus-located. Gene structure analysis showed that *VcAN1* gDNA contained eight introns, while all the others contained seven introns. Many light-, phytohormone-, stress- and plant growth and development-related *cis*-acting elements and transcription factor binding sites (TFBSs) were identified in their promoters, but the types and numbers of *cis*-elements and TFBSs varied greatly between the two *bHLH* groups. Quantitative real-time PCR results showed that *VcAN1* expressed highly in old leaf, stem and blue fruit, and its expression increased as the blueberry fruit ripened. Its expression in purple podetium and old leaf was respectively significantly higher than in green podetium and young leaf, indicating that *VcAN1* plays roles in anthocyanin biosynthesis regulation not only in fruit but also in podetium and leaf. *VcbHLH1-1* expressed the highest in young leaf and stem, and the lowest in green fruit. The expression of *VcbHLH1-1* also increased as the fruit ripened, and its expression in blue fruit was significantly higher than in green fruit. *VcbHLH1-2* showed high expression in stem but low expression in fruit, especially in red fruit. Our study indicated that the anthocyanin biosynthesis regulatory functions of these *bHLHs* showed certain spatiotemporal specificity. Additionally, *VcAN1* might be a key gene controlling the anthocyanin biosynthesis in blueberry, whose function is worth exploring further for its potential applications in plant high anthocyanin breeding.

## 1. Introduction

Anthocyanins, a kind of natural polyphenols widely exist in many plants, are known to have great health-promoting effects mostly due to their high antioxidant activity. In recent years, a large number of studies on anthocyanin metabolism have been carried out [1,2]. To explore the anthocyanin biosynthesis mechanism, many structural genes, such as *PAL*, *CHS*, *DFR*, *ANS*, *F3H* and *CHI* encoding key enzymes directly catalyzing anthocyanin biosynthesis have been isolated and functionally identified. Many transcription factors have also been proved to contribute to the anthocyanin biosynthesis regulation. Among them, the MBW complex, composed of R2R3-MYB protein, bHLH protein of MYC family and transcription factors of the WD40 protein family, is believed to play a key role in regulating tissue specific expression of typical anthocyanins [3,4]. They can bind to the promoter regions of one or more anthocyanin biosynthesis structural genes, and lead to the activation or inhibition of the expression of their corresponding targets [5,6].

The bHLH transcription factors comprise the second largest plant transcription factor superfamily, and many members have been inferred to play roles in anthocyanin biosynthesis [7,8]. The Lc protein encoded by the maize *R* gene was the first bHLH identified in plants, which could regulate at least two anthocyanin biosynthesis structural genes [9]. Its overexpression in *Arabidopsis thaliana* and tobacco have been reported to have the ability to improve anthocyanin accumulations [10]. Arabidopsis bHLH transparent testa 8 (TT8), a homologous protein of maize R protein, was reported to be necessary for *DFR* and *BAN* gene expression and plays an important role in controlling flavonoid metabolism in Arabidopsis seed coat [11]. Lotus *NnTT8* is a homologous gene of *AtTT8*, and its overexpression can restore the anthocyanin and proanthocyanin accumulation of the *AtTT8* mutant [12]. The up-regulated expression of anthocyanin synthesis-related *BjTT8* in *Brassica juncea* contributed greatly to the formation of purple leaves [13]. The expression of apple *MdbHLH3* gene is low temperature inducible, and its encoded protein interacts with MdMYB1, a transcription factor controlling apple anthocyanin biosynthesis. In addition, MdbHLH3 can bind to promotors of *MdDFR*, *MdUFGT* and *MdMYB1* to regulate their expression, and play an important role in anthocyanin accumulation and fruit coloring triggered by low temperature [14]. Citrus Neomi and PH4 form a regulatory complex for proanthocyanidin synthesis. The overexpression of *Neomi* activated the expression of proanthocyanidin synthesis-related genes and improved proanthocyanidin content in citrus callus [15,16]. The foxtail millet PPLS1 could interact with SiMYB85 to regulate the anthocyanin biosynthesis, and transient co-expression of PPLS1 and SiMYB85 in tobacco leaves resulted in anthocyanin accumulation and up-regulated expression of anthocyanin synthesis genes in tobacco leaves [17]. *ThMYC4E* is the candidate *bHLH* gene controlling the blue grain trait from *Th. Ponticum*, its overexpression in common wheat JW1 activated the anthocyanin biosynthesis in transgenic plants and led to much higher anthocyanin accumulation in grains, leaves and glumes [18]. The Petunia bHLH protein AN1 was reported to be involved in the tomato anthocyanin regulation by directly activating the expression of *DRF* and *MYB* genes [19]. All the above-mentioned reports suggest that bHLH proteins play a key role in anthocyanin biosynthesis in plants.

Blueberry (*Vaccinium corymbosum* L.) is native to North America and East Asia. It has only been cultivated for a little more than 100 years, but its annual yield ranks only second to strawberry among all the berries. Blueberries are rich in nutrients, especially anthocyanins [20], which makes blueberry an ideal material for studying the anthocyanin biosynthesis and regulation metabolism in fruits. Due to the rapid development of molecular biology and high throughput sequencing techniques, many blueberry anthocyanin biosynthesis-related structural genes have been identified [21,22]. Additionally, much attention has been paid to the blueberry anthocyanin synthesis-related *MYB* genes [23,24,25,26,27,28]. Compared to MYB transcription factors, however, research focusing on the blueberry anthocyanin biosynthesis related bHLHs has been much less. In addition, the reported anthocyanin biosynthesis related bHLH proteins or genes were mostly screened based on proteome or transcriptome data. For example, according to their proteome and metabolome profiling data, Li et al. found that the expression of a VcbHLH3 protein (CUFF.37765.1) in the pink fruit was significantly higher than that in the blue fruit, and speculated that it might play a regulatory role in the synthesis of anthocyanins/flavonoids in blueberry [29]. Zhao et al. identified seven candidate bHLHs by blasting homologous MBW proteins of Arabidopsis, apple, grape and strawberry against their blueberry transcriptomic data, and found that the VcbHLH1 protein could interact with the VcMYBL1, and thus contributed to the blueberry anthocyanin biosynthesis regulation [1].

The publications of the draft blueberry genome data [30,31] will undoubtedly facilitate the identification and characterization of blueberry anthocyanin biosynthesis-related genes and the exploration of the regulation networks of the anthocyanin metabolism [32]. In this study, based on the blueberry genome data, anthocyanin biosynthesis-related bHLHs were identified through homologous protein sequence alignment under strict criteria using reported anthocyanin-related bHLHs from several plant species, including Arabidopsis, apple, kiwifruit, citrus, eggplant, and so on. Their gene and protein sequences were then bioinformatically characterized. Moreover, to explore their regulatory roles in different organs including fruits at different developmental stages, the expression patterns of these identified *bHLHs* were examined using quantitative real-time reverse transcription PCR (qRT-PCR). The results obtained in this study will provide basis for the future function analysis of bHLH transcription factors and for research and applications of *bHLH* genes in high anthocyanin blueberry breeding.

## 2. Results

### 2.1. The Identified Anthocyanin Biosynthesis-Related Blueberry bHLH Genes

**Figure 1:**
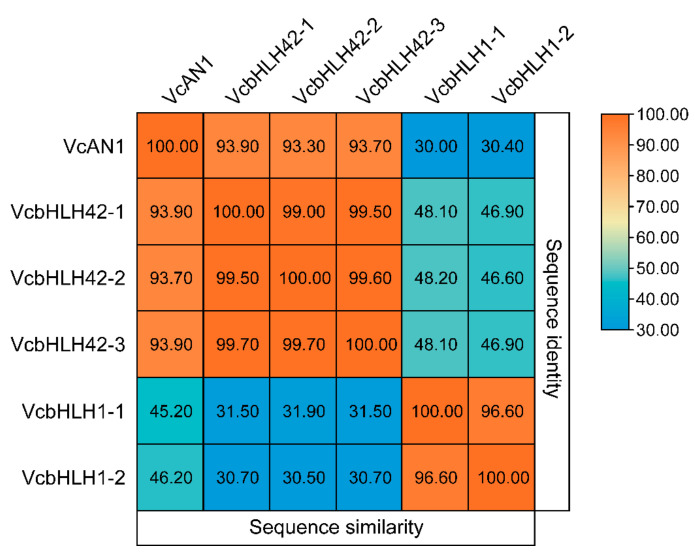
Sequence identities and similarities (%) among the six candidate anthocyanin biosynthesis-related blueberry bHLH proteins.

In total, six candidate blueberry anthocyanin biosynthesis-related bHLH proteins were identified from blueberry genome data through homologous protein sequence alignment using reported anthocyanin biosynthesis-related bHLHs from Arabidopsis, apple, kiwifruit, and some other plant species [11,13,14,15,16,17,18,33,34,35,36,37,38,39]. According to their homologous protein names, they were named as VcAN1 (VaccDscaff11-processed-gene-379.7), VcbHLH42-1 (VaccDscaff24-augustus-gene-24.28), VcbHLH42-2 (VaccDscaff15-augustus-gene-371.25), VcbHLH42-3 (VaccDscaff19-augustus-gene-381.30), VcbHLH1-1 (VaccDscaff28-augustus-gene-45.27) and VcbHLH1-2 (VaccDscaff44-augustus-gene-0.19), respectively (Table 1). By analyzing their protein similarities, it was found that similarities among VcAN1, VcbHLH42-1, VcbHLH42-2 and VcbHLH42-3 were all very high (>93%), but their protein similarities with VcbHLH1-1 and VcbHLH1-2 were all less than 32%. In addition, the similarity between VcbHLH1-1 and VcbHLH1-2 was higher than 96% (Figure 1). This indicated that the six blueberry bHLHs could be divided into two groups.

The eggplant SmelAN1 (Smel_009G3266401.01) shared the highest similarity with VcAN1, of up to 77.12%. The similarities of kiwifruit AcbHLH42 (MH643775), apple MdbHLH3 (ADL36597.1) and citrus Neomi (Cs5g31400) with VcAN1 were all higher than 60%, which was 74.42%, 65.08% and 63.08%, respectively. The similarity of AcbHLH42 with VcbHLH42-1, VcbHLH42-2 and VcbHLH42-3 was 79.10%, 79.37% and 79.23%, respectively. Arabidopsis AtTT8 (At4G09820), apple MdbHLH3 (ADL36597.1) and citrus Neomi (Cs5g31400) all shared more than 60% similarity with VcbHLH42-1, which was 73.21%, 69.03% and 67.25%, respectively. Apple MdbHLH3 (ADL36597.1) and citrus Neomi (Cs5g31400) shared more than 60% similarity with both VcbHLH42-2 and VcbHLH42-3. *Populus alba* PalbHLH1 (PAYT030711.1) shared the highest similarity with VcbHLH1-1 and VcbHLH1-2 (57.12% and 55.61%, respectively). The similarity between VcbHLH1-1 and Petunia PPLS1 (SeITa.7G195400) was 57.12%, and the similarity of VcbHLH1-1 with SmelJAF13 (Smel_008G3192001.01), AtEGL3 (At1G63650), AtGL3 (At5G41315) and ThMYC4E (KX914905) was 53.85%, 48.23%, 47. 77% and 37.54%, respectively. Moreover, the similarity of VcbHLH1-2 with eggplant SmelJAF13, Arabidopsis AtEGL3 and AtGL3 was 48.55%, 46.95% and 46.42%, respectively. The large similarities between these blueberry bHLHs and bHLHs from other plant species indicated that they might have similar functions as their corresponding homologous proteins.

### 2.2. Physiochemical Properties Analysis of Blueberry bHLH Proteins

The CDS length of the six blueberry bHLH genes ranges from 1803 bp to 2328 bp, and their encoded proteins contain 600~775 aa, with molecular weight ranging from 67,581.48 to 85,474.08 Da, and the isoelectric point (PI) ranging from 5.45 to 6.25. According to the PI, instability index and grand average of hydropathicity (GRAVY) values, all thes VcbHLHs were predicted to be acidic, unstable hydrophilic proteins (Table 2). Subcellular localization prediction results showed that all the six blueberry bHLHs were nucleus-localized (Table 2).

### 2.3. Gene Structrues of VcbHLH Genes and Conserved Conmains and Motifs in Their Encoded Proteins

**Figure 2:**
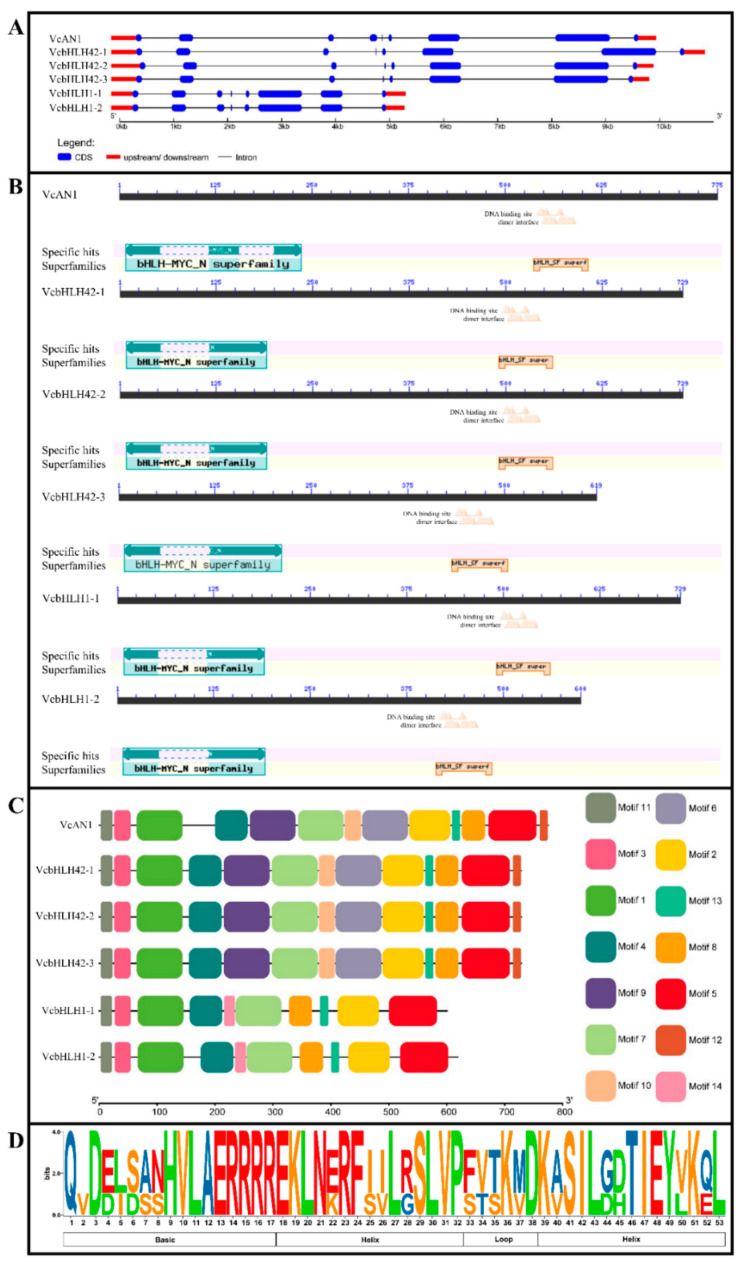
Gene structures of VcbHLH genes and conserved domain and motif distributions in their encoded proteins. (**A**) Gene structure; (**B**) Conserved domains; (**C**) Conserved motifs; (**D**) Sequence logo for the bHLH domain.

By analyzing the structures of the six blueberry *bHLH* genes, it was found that *VcAN1* gDNA contained eight introns, while other *bHLH* genes contained only seven introns. Moreover, the total intron lengths of *VcbHLH1-1* and *VcbHLH1-2* were found to be much shorter than the other four blueberry *bHLHs* (Figure 2A).

CDD verification revealed that all these blueberry bHLHs contained bHLH-MYC_N domain (PF14215.5) and bHLH_SF super domain. In addition, the N-terminus of bHLH_SF super domain of all the bHLH proteins contained DNA binding sites and dimerization interfaces (Figure 2B).

Conserved motif analysis showed that VcAN1 and VcbHLH42-1~3 contained a total of 13 motifs (Motif1~Motif13). VcbHLH1-1 and VcbHLH1-2 did not contain Motif6, Motif9 and Motif10, but they contained an extra Motif14 (Figure 2C). Motif2 contains most of the bHLH domain. Conversation analysis of the bHLH domain showed that Asp-3, His-9, Val-10, Ala-11, Glu-12, Arg-13, Arg-14, Arg-15, Arg-16 and Arg-17 in the basic region, Glu-18, Lys-19, Leu-20, Asn-21, Arg-23, Phe-24, Leu-27, Ser-29, Leu-30, Val-31 and Pro-32 in the helix 1 region, Lys-36 in the loop region, and Asp-38, Lys-39, Ser-41, Ile-42, Leu-43, Thr-46, Ile-47, Glu-48, Val-49, Lys-51 and Leu-53 in the helix 2 region were highly conserved among all the six blueberry bHLHs (Figure 2D).

### 2.4. Phylogenetic Analysis of Blueberry Anthocyanin Biosynthesis-Related bHLHs

**Figure 3:**
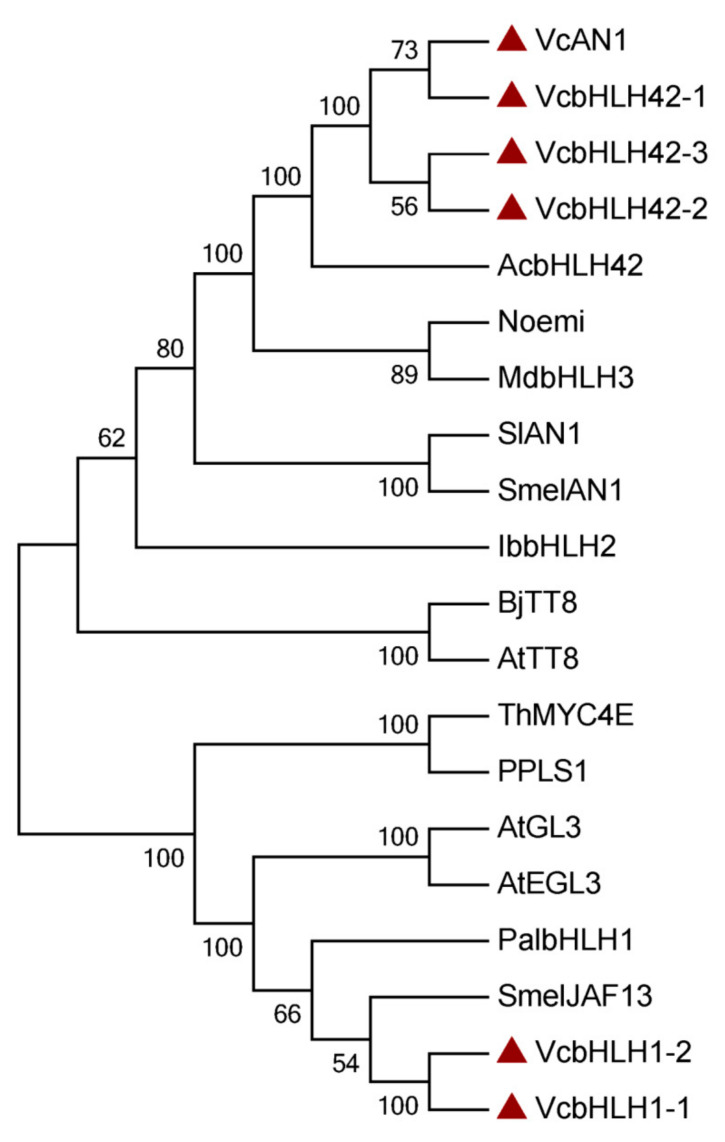
Phylogenetic tree constructed using anthocyanin biosynthesis-related bHLHs from blueberry and some other plants. SmelAN1: SMEL_009g326640.1.01; AcbHLH42: MH643775; MdbHLH3: ADL36597.1; Noemi: Cs5g31400; SlAN1: KR076778; IbbHLH2: itf14g18730.t2; AtTT8: At4G09820; BjTT8: BjuB004115; PalbHLH1: PAYT030711.1; PPLS1: Seita.7G195400; SmelJAF13: SMEL_008g319200.1.01; AtEGL3: At1G63650; AtGL3: At5G41315; ThMYC4E: KX914905; VcAN1: VaccDscaff11-processed-gene-379.7; VcbHLH42-1: VaccDscaff24-augustus-gene-24.28; VcbHLH42-2: VaccDscaff15-augustus-gene-371.25; VcbHLH42-3: VaccDscaff19-augustus-gene-381.30; VcbHLH1-1: VaccDscaff28-augustus-gene-45.27; VcbHLH1-2: VaccDscaff44-augustus-gene-0.19.

Phylogenetic analysis showed that the six blueberry anthocyanin biosynthesis-related bHLHs could be divided into two groups, which was consistent with the results of protein similarity, gene structure, and conserved motif analysis. VcAN1, VcbHLH42-1, VcbHLH42-2 and VcbHLH42-3 were clustered into one group, and their relationship with kiwifruit AcbHLH42 was the closest. VcbHLH1-1 and VcbHLH1-2 were clustered into the other group, and they showed close relationship with SmelJAF13 and PalbHLH1 (Figure 3).

### 2.5. Chromosomal Location Analysis of Blueberry Anthocyanin Biosynthesis-Related bHLH Genes

**Figure 4:**
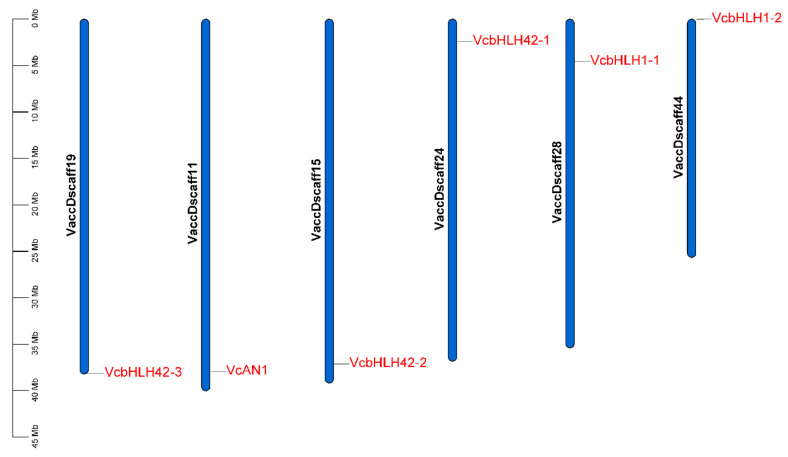
Chromosomal location of the six blueberry anthocyanin biosynthesis-related *bHLH* genes. VcAN1: VaccDscaff11-processed-gene-379.7; VcbHLH42-1: VaccDscaff24-augustus-gene-24.28; VcbHLH42-2: VaccDscaff15-augustus-gene-371.25; VcbHLH42-3: VaccDscaff19-augustus-gene-381.30; VcbHLH1-1: VaccDscaff28-augustus-gene-45.27; VcbHLH1-2: VaccDscaff44-augustus-gene-0.19.

Chromosomal location analysis of the six *VcbHLH* genes showed that they were separately located on six different scaffolds. *VcAN1*, *VcbHLH42-1*, *VcbHLH42-2*, *VcbHLH42-3*, *VcbHLH1-1* and *VcbHLH1-2* was located on Vaccdscaffolds11, 24, 15, 19, 28 and 44, respectively (Figure 4).

### 2.6. cis-Acting Elements and TFBSs Distribution in Promoters of Blueberry Anthocyanin Biosynthesis-Related bHLH Genes

**Figure 5:**
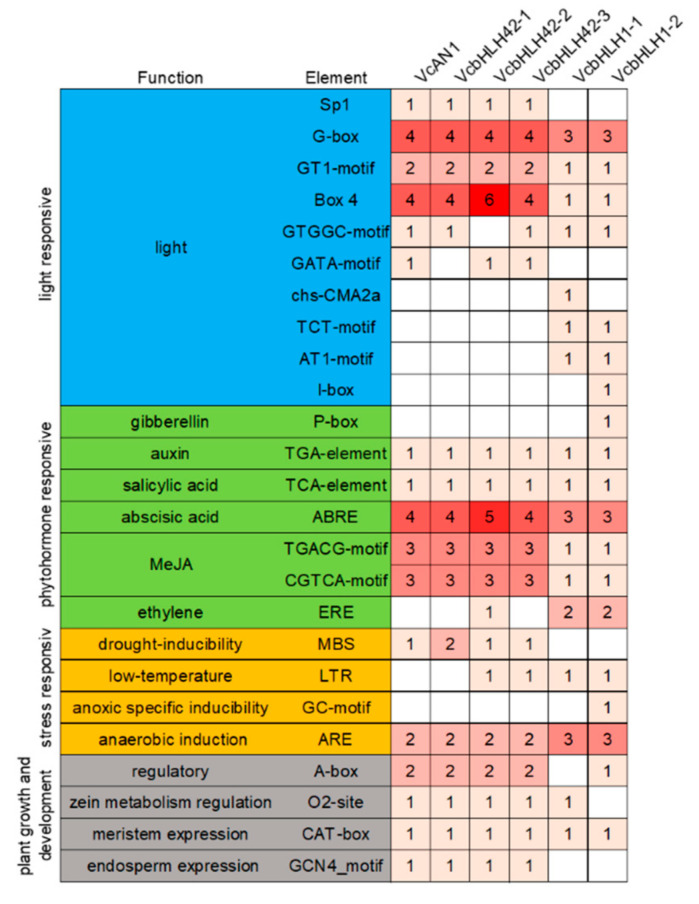
The identified *cis*-acting elements in promoters of anthocyanin biosynthesis-related *VcbHLHs*.

The distribution of *cis*-acting elements in the promoters of the six *bHLH* genes were analyzed. Result showed that the types and numbers of *cis*-acting elements in promoters of *bHLH* genes belonging to the same group were much more similar (Figure 5). These *cis*-acting elements can be further classified into four categories: light response-, plant hormone response-, stress response- and plant growth and development-related [40], each including 10, 7, 4 and 4 *cis*-acting elements, respectively. Among them, the types and numbers of light response-related elements differed greatly. For example, the Sp1 element was found in promoters of *VcAN1*, *VcbHLH42-1*, *VcbHLH42-2* and *VcbHLH42-3*, but not in the promoters of *VcbHLH1-1* and *VcbHLH1-2*; the numbers of G-box, GT1-motif and BOX-4 in promoters of *VcAN1*, *VcbHLH42-1*, *VcbHLH42-2* and *VcbHLH42-3* were greater than that of *VcbHLH1-1* and *VcbHLH1-2* promoters; *VcAN1*, *VcbHLH42-1*, *VcbHLH42-2* and *VcbHLH42-3* promoters did not contain chS-CMA2a, TCT-Motif, AT1-motif and I-box elements that existed in the promoters of *VcbHLH1-1* and/or *VcbHLH1-2*.

Many auxin-, salicylic acid (SA)-, abscisic acid (ABA)-, methyl jasmonate (MeJA)- and some other hormone-responsive elements were identified in the six *bHLH* genes’ promoters. The number of ABA- and MeJA-responsive elements in *VcAN1*, *VcbHLH42-1*, *VcbHLH42-2* and *VcbHLH42-3* promoters were all larger than that in *VcbHLH1-1* and *VcbHLH1-2* promoters. The *VcbHLH42-2* promoter contained the largest amount of ABRE ABA-responsive elements (five in total). The *VcbHLH1-2* promoter specifically contained a gibberellin-responsive element p-box. Moreover, the promoter of *VcbHLH42-2*, *VcbHLH1-1* and *VcbHLH1-2* respectively contained 1, 2 and 2 ethylene response element EREs, while the other three genes’ promoters did not contain this element.

Except *VcbHLH1-1* and *VcbHLH1-2*, the promoters of all the other four genes contained the drought induction response element MBS, and the *VcbHLH42-1* promoter contained two of the elements. The promoters of *VcbHLH42-2*, *VcbHLH42-3*, *VcbHLH1-1* and *VcbHLH1-2* each contained one low temperature response element, LTR. The VcbHLH1-2 promoter specifically contained a GC-motif specific hypoxia induction-related element. All the promoters contained anaerobic induction-related element ARE, and promoters of *VcAN1*, *VcbHLH42-1*, *VcbHLH42-2* and *VcbHLH42-3* contained two of this element, while the other two genes’ promoters contained three.

For the plant growth and development-related elements, all promoters contained the meristem expression element, CAT-box. *VcAN1*, *VcbHLH42-1*, *VcbHLH42-2* and *VcbHLH42-3* promoters contained the same types and amounts of A-box, O2-site and GCN4_motif, while VcbHLH1-1 promoter had no A-box and GCN4_motif elements and the *VcbHLH1-2* promoter had no O2-site and GCN4_motif components.

**Figure 6:**
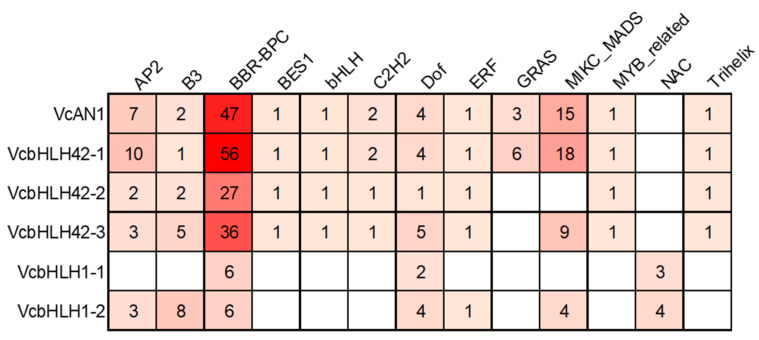
Transcription factor binding sites predicted in the promoters of anthocyanin biosynthesis-related *VcbHLHs*.

The transcription factor binding sites (TFBS) prediction result showed that there were binding sites for 13 kinds of transcription factors, including AP2/ERF, B3, BFR-BPC, BES1, bHLH, C2H2, Dof, ERF, GRAS, MIKC_MADS, MYB_related, NAC and Trihelix, in the promoters of the six blueberry *bHLH* genes (Figure 6). The types and numbers of TFBSs on each gene’s promoter varied greatly. There were 12 types of TFBSs on *VcAN1* and *VcbHLH42-1* promoters, 10 types of TFBSs on *VcbHLH42-2* promoter, 11 types of TFBSs on *VcbHLH42-3* promoter, 7 types of TFBSs on *VcbHLH1-2* promoter, but only 3 types of TFBS on *VcbHLH1-1* promoter. Binding sites for BBR-BPC and Dof transcription factors were found on all the promoters of the six blueberry *bHLHs*, and the promoters of *VcAN1*, *VcbHLH42-1*, *VcbHLH42-2* and *VcbHLH42-3* contained significantly more BFR-BPC binding sites than the others. Binding sites for BES1, bHLH, C2H2, MYB-MADS and Trihelix transcription factors were found only on the promoters of *VcAN1*, *VcbHLH42-1*, *VcbHLH42-2* and *VcbHLH42-3*. In addition, NAC binding sites existed only on *VcbHLH1-1* and *VcbHLH1-2* promoters. Moreover, GRAS binding sites existed only on *VcAN1* and *VcbHLH42-1* promoters.

### 2.7. Protein-Protein Interaction Analysis of Blueberry bHLH Proteins

**Figure 7:**
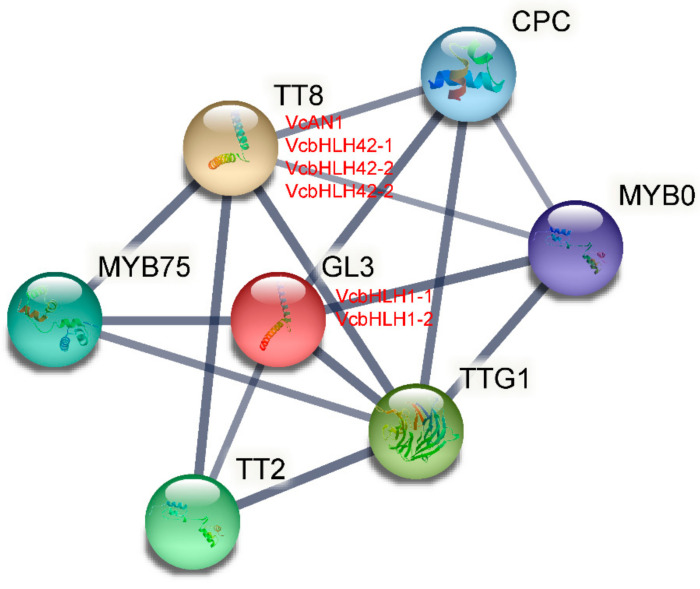
Protein–protein interaction network for blueberry anthocyanin biosynthesis-related VcbHLHs according to bHLHs orthologs in Arabidopsis.

Based on the Arabidopsis protein database, the protein–protein interactions of the six blueberry bHLHs were predicted using STRING software. As shown in Figure 7, VcAN1, VcbHLH42-1, VcbHLH42-2 and VcbHLH42-3 were identified to be homologous proteins of Arabidopsis TT8 (At4G09820), while VcbHLH1-1 and VcbHLH1-2 were defined as homologous proteins of Arabidopsis GL3 protein (At5G41315). In addition, some well-known anthocyanin-related proteins, including MYB75 (At1G56650), TT2 (At5G35550), TTG1 (At5G24520), MYB0 (At3G27920) and CPC (At2G46410), were predicted to have the ability to interact with these blueberry bHLH proteins.

### 2.8. Cloning and Expression Analysis of Blueberry Anthocyanin Biosynthesis-Related bHLH Genes

**Figure 8:**
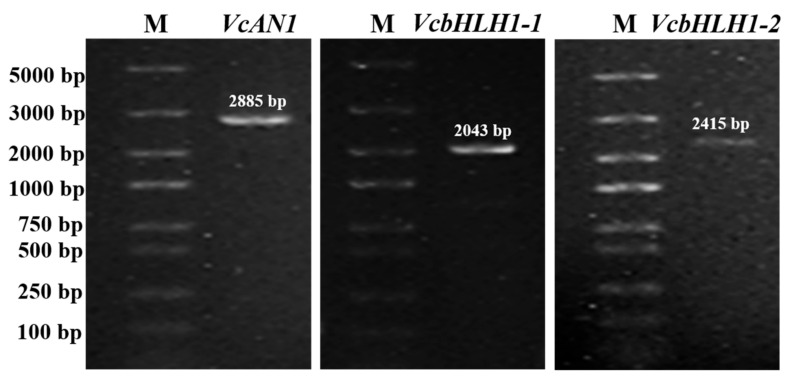
Electrophoresis detection results for PCR products of blueberry anthocyanin biosynthesis-related *VcAN1*, *VcbHLH1-1* and *VcbHLH1-2* genes. M: DL5000 marker.

Given that the blueberry *bHLHs* belonging to the same group shared very high similarity, in this study, gene specific primers for three of the six *bHLH* genes were successfully designed according to their nucleotide sequences deposited in the blueberry genome data hub. By using gene-specific primers, cDNA sequences of *VcAN1*, *VcbHLH1-1* and *VcbHLH1-2*, respectively with lengths of 2885 bp, 2043 bp and 2415 bp, were amplified (Figure 8). Sequencing results confirmed that these genes were successfully amplified.

**Figure 9:**
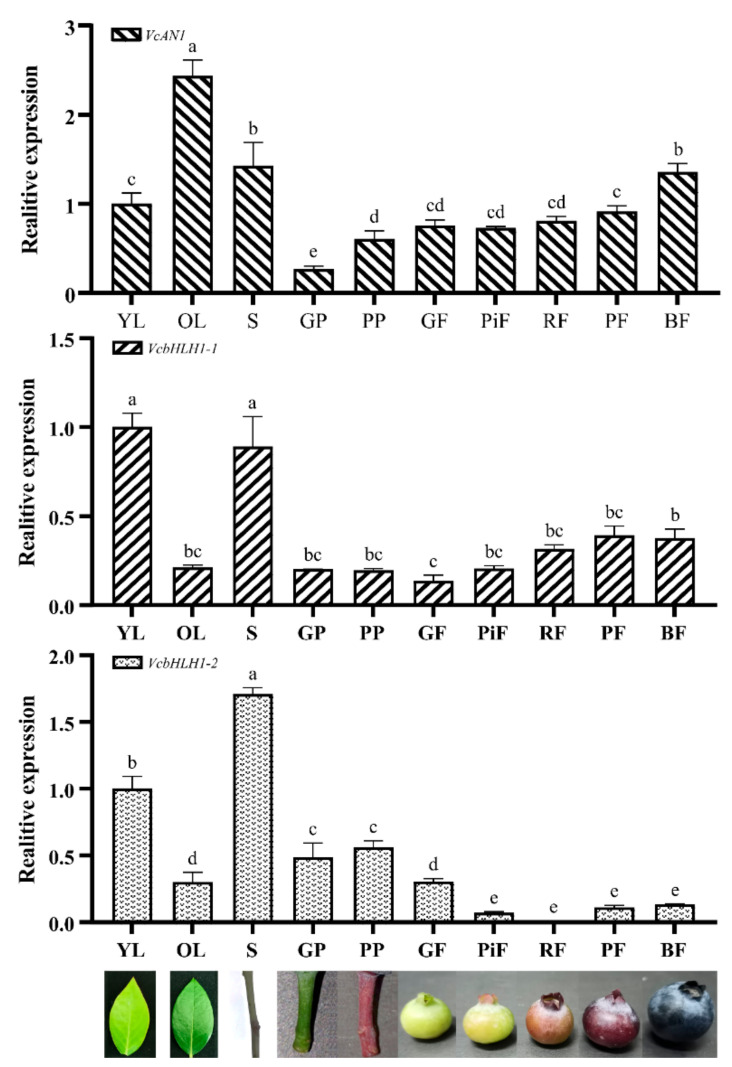
Relative expression of *VcAN1*, *VcbHLH1-1* and *VcbHLH1-2* in blueberry young leaf (YL), old leaf (OL), stem (S), green podetium (GP), and purple podetium (GP), and fruits at green (GF), pink (PiF), red (RF), purple (PF), and blue (BF) developmental stages. Different letters above the columns represent significant difference among samples (*p* < 0.01).

To reveal their expression patterns in young leaf (YL), old leaf (OL), stem (S), green podetium (GP) and purple podetium (PP) and fruits at green (GF), pink (PiF), red (RF), purple (PF) and blue (BF) developmental stages, qRT-PCR experiments were performed. Results showed that the expression of *VcAN1*, *VcbHLH1-1* and *VcbHLH1-2* varied greatly in different organs and in fruits at different developmental stages (Figure 9). *VcAN1* showed the highest expression in OL and the lowest expression in podetium. Its expression in OL was about 2.43 times of that in YL, and about 9.12 times of that in GP. The *VcAN1* expression in GP and PP also differed significantly, and its expression in PP was about 2.27 times of that in GP. The *VcAN1* expression in S and BF ranked the second and the third, respectively. In fruits at different developmental stages, the *VcAN1* expression level follows the order: BF > PF > RF > GF > PiF, and its expression in BF is about 1.86 and 1.79 times of that in PiF and GF, respectively. Unlike the *VcAN1*, the highest expression of *VcbHLH1-1* was found in YL and S. Its expression in YL was about 4.73 times of that in OL. *VcbHLH1-1* expressed the lowest in GF (accounting for about 13.7% of YL), and its expression in PF and BF was significantly higher than that in GF, which was about 2.86 times and 2.75 times compared to GF, respectively. The expression of *VcbHLH1-1* in GP and PP was similar, both accounting for about 20% of YL. *VcbHLH1-2* showed the highest expression in S and the lowest expression in RF, and its expression in S was about 206 times of that in RF. Its expression in YL is about 58.7% in S, but is about 3.32 times of that in OL. Its expression in PP was higher than in GP, and its expression in the two kinds of podetiums are both significantly higher than that in fruits. The expression level of *VcbHLH1-2* in fruits at different developmental stages followed the order: GF > BF > PF > PiF > RF. In addition, its expression in GF was about 4.24, 36.94, 2.72 and 2.29 times of that in PiF, RF, PF and BF, respectively.

In our previous study, we measured the contents of total phenolic, falvonoid and anthocyanin of blueberry fruits at the same five different developmental stages [16]. To uncover the roles of *bHLH* genes in secondary metabolites biosynthesis in blueberry fruits, the correlation between the *bHLH* genes’ expression and secondary metabolites contents in blueberry fruits were investigated. Results showed that *VcAN1* expression level was very significantly negatively correlated with total phenolic content and flavonoid content (correlation coefficient was −0.730 and −0.717, respectively), and very significantly positively correlated with anthocyanin content (correlation coefficient was 0.940). The expression level of *VcbHLH1-1* was very significantly negatively correlated with contents of total phenolic and flavonoids (correlation coefficient was −0.717 and −0.732, respectively), and somewhat positively correlated with anthocyanin content (correlation coefficient was 0.533). However, the expression level of *VcbHLH1-2* was significantly positively correlated with total phenolic content and flavonoid content (correlation coefficient was 0.647 and 0.569, respectively), and slightly negatively correlated with anthocyanin content (correlation coefficient was 0.144).

## 3. Discussion

Blueberry is popular and famous for its anthocyanin-rich characteristics. However, as a young fruit tree species that has been cultivated for only a little more than 100 years, research on the molecular mechanism of blueberry anthocyanin biosynthesis and regulation are very limited. Recently, the regulation roles of several blueberry MYB transcription factors have been clarified [7,8,9,10,27,28]. However, as an important part of the MBW complex regulating anthocyanin biosynthesis, the blueberry bHLH transcription factors were paid much less attention. In this study, we identified six bHLH proteins based on the blueberry genome data by homologous protein alignment screening.

### 3.1. The Identified Blueberry Anthocyanin Biosynthesis-Related bHLHs Contained Conserved bHLH Domains and Key Amino Acids Required for Their DNA Binding Activity and Functions

All the six bHLH proteins contained the conserved bHLH-MYC_N domain (PF14215.5), the bHLH_SF super domain, DNA binding sites and dimerization interfaces. The length of the conserved bHLH domain usually contained approximately 50~60 amino acids [41], and the conserved domain for these blueberry bHLHs reported in this study was identified to consist of 53 amino acids. Previous studies indicated that the conserved His-9, Glu-13, Arg-16 and Arg-17 in the basic region of the bHLH sequence are necessary for DNA binding, the conserved Leu-29 and Leu-65 in the helix region are of great significance for bHLH dimerization activity, and the conserved Lys-36 in the loop region is highly conserved [42,43,44]. Consistently, the six VcbHLHs identified in this study all possess these conserved amino acids. According to previous classification criteria, they all belonged to G-box binding proteins [45,46].

### 3.2. Blueberry Anthocyanin Biosynthesis-Related bHLHs Could Be Classified into Two Groups, and the Functions of Each Group Differed from Each Other

According to the results of protein phylogenetic analysis, the six blueberry bHLHs could be further divided into two groups. One group included VcAN1, VcbHLH42-1, VcbHLH42-2 and VcbHLH42-3. This group of VcbHLHs showed the highest similarity to well-known anthocyanin biosynthesis-related eggplant SmelAN1 [33] or kiwifruit AcbHLH42 [34]. Moreover, they were all identified as homologous proteins of Arabidopsis AtTT8 [11]. *SmelAN1* was expressed in all eggplant tissues containing anthocyanins, and showed the highest expression level in fruits [33]. The kiwifruit *AcbHLH42*, also a homologous gene of *AtTT8*, was highly expressed in the inner pericarp of kiwifruit with the highest anthocyanin content, and its encoding protein was necessary for the activation of *AcANS* and *AcF3GT* promoters. The co-expression of AcMYB123 and AcbHLH42 was reported to be an important prerequisite for anthocyanin synthesis, indicating that AcbHLH42 is a key factor in the spatiotemporal regulation of anthocyanin synthesis in kiwifruit [34]. Arabidopsis TT8, a homologous protein of maize R protein, is required for the expression of anthocyanin biosynthesis structural genes, *DFR* and *BAN* [11]. The other group of VcbHLHs consisted of two members, VcbHLH1-1 and VcbHLH1-2. This group of VcbHLHs showed the highest similarity to *Populus alba* PalbHLH1 and was closely related to SmeJAF13 and PalbHLH1; they were also identified as homologous proteins of Arabidopsis AtGL3. The overexpression of the *PalbHLH1* gene in poplar enhanced the pathogen resistance of transgenic poplar, and this effect was reported to be achieved by increasing the flavonoid accumulation [38]. Similar to *SmelAN1*, *SmelJAF13* was also expressed in all eggplant tissues containing anthocyanins, but it showed the highest expression level in flower organs [33]. Moreover, the Arabidopsis AtGL3 has been reported to function in specifying the root epidermal cell fate [39]. It was suggested that the blueberry bHLHs belonging to different groups might function in different organs or tissues. Our qRT-PCR result also confirmed that the expression levels of blueberry *bHLH* genes in different organs varied greatly. *VcAN1* showed high expression in OL, S and BF. Its expression in fruit increased as the blueberry fruit ripened and was very significantly positively correlated with the anthocyanin content in fruit, implying that it regulates the anthocyanin biosynthesis in blueberry fruit. Moreover, it was also worth noting that its expression in OL was significantly higher than in YL. The expression level of *VcAN1* in podetium was the lowest. Notably, its expression in PP was significantly higher than in GP. This evidence indicated that *VcAN1* might play important positive roles in the anthocyanin accumulation in different blueberry organs. *VcbHLH1-1* expressed the highest in YL and S, and the lowest in GF. Its expression in YL was significantly higher than in OL. Moreover, the expression of *VcbHLH1-1* also increased as the fruit ripened, and its expression in BF was significantly higher than in GF. Similar to *VcbHLH1-1*, *VcbHLH1-2* showed high expression in YL and S, and its expression in YL was also significantly higher than in OL, suggesting that *VcbHLH1-1* and *VcbHLH1-2* might play similar roles in blueberry leaf and stem. Unlike *VcbHLH1-1*, however, *VcbHLH1-2* showed very low expression in blueberry fruit, and its expression in fruits at different developmental stages followed the order: GF > BF > PF > PiF > RF, suggesting that their functions in blueberry fruit varied. Thus, it was indicated that the anthocyanin biosynthesis regulatory roles of these *VcbHLHs* were spatially and temporally different [19,33,47].

### 3.3. There Are Many Factors Influencing the Expression and Functions of VcbHLH Genes

The expression of *bHLHs* can be affected by many envrionmental fators, transcription factors and so on [48]. The anthocyanin biosynthesis and accumulation are greatly influenced by light [49]. In this study, we found that the types and amounts of light-responsive elements in promoters of the six anthocyanin biosynthesis-related blueberry *bHLHs* varied greatly between the two groups. This suggested that the light responses of *bHLHs* belonging to different groups varied.

The anthocyanin biosynthesis and accumulation are also greatly influenced by phytohormones [48,49,50]. In the present study, we identified a large number of hormone-responsive elements in promoters of the six *bHLH* genes. Consistent with the protein phylogenetic analysis result, the hormone-responsive element types and numbers in promoters of blueberry *bHLHs* belonging to the different groups varied greatly. Accumulated evidence has proved that many bHLHs display functions via influencing ABA- and JA- signaling pathways [51,52]. In addition, ABA and JA can both influence the anthocyanin accumulation in plants [53,54]. Consistently, in this study, the numbers of ABA- and MeJA-responsive elements in these six blueberry *bHLH* promoters were the largest among all hormone-responsive elements. It was thus indicated that these two hormones greatly affected the anthocyanin accumulation by regulating *bHLH* expression in blueberries. The numbers of ABA- and MeJA-responsive elements in promoters of *VcAN1*, *VcbHLH42-1*, *VcbHLH42-2* and *VcbHLH42-3* were greater than the other two genes’ promoters. Interestingly, we identified two ERE elements in promoters of *VcbHLH1-1* and *VcbHLH1-2*, indicating that these two *bHLHs* might play their roles through responding to or regulating ethylene signaling [55]. Moreover, their expression in OL were both significantly lower than in YL, suggesting that they might play some roles in leaf development and senescence.

*bHLH* genes have been increasingly proved to be involved in plant abiotic stress responses, including osmotic stress, drought, low temperature and so on [48]. All the promoters of these blueberry *bHLHs* contained anaerobic induction-related elements, and the *VcbHLH1-2* promoter specially harbored an anoxic specific inducibility-related element, indicating that these *bHLHs* might all be osmotic stress responsive. The promoters of *VcAN1*, *VcbHLH42-1*, *VcbHLH42-2* and *VcbHLH42-3* contained drought inducibility-related elements that did not exist in promoters of members belonging to the other group, indicating that the expression of two blueberry *bHLH* groups differed in response to drought. Moreover, the promoter of *VcbHLH42-2*, *VcbHLH42-3*, *VcbHLH1-1* and *VcbHLH1-2* each contained a low-temperature-related element, LTR, suggesting that their expression was regulated by low temperature.

Transcription factors also greatly influenced the transcriptional activities of bHLHs [56]. Many bHLHs have been identified to harbor MYB binding sites [48]. In our present study, MYB-related binding sites were only predicted in promoters of *VcAN1*, *VcbHLH42-1*, *VcbHLH42-2* and *VcbHLH42-3*. *VcbHLH1-1* and *VcbHLH1-2* promoters, however, specifically possess several binding sites for NAC transcription factors. In sweet potato, a MYB340-bHLH2-NAC56 complex was reported to regulate anthocyanin biosynthesis [37]. Similarly, a *NAC* gene was identified to be involved in anthocyanin accumulation in blueberry [25]. Therefore, it is reasonable to hypothesize that the VcbHLH1-1 and VcbHLH1-2 might interact with NAC transcription factors to regulate the synthesis of anthocyanin. However, this hypothesis still needs to be further verified. Moreover, binding sites for bHLH, BES1, C2H2, MYB-MADS and Trihelix transcription factors were only found in the promoters of *VcAN1*, *VcbHLH42-1*, *VcbHLH42-2* and *VcbHLH42-3*, indicating that the expression and function of different blueberry *bHLH* groups was regulated by different transcription factors.

## 4. Materials and Methods

### 4.1. Plant Materials

The young leaf (YL), old leaf (OL), stem (S), green podetium (GP), and purple podetium (PP), and fruits at green (GF), pink (PiF), red (RF), purple (PF), and blue (BF) developmental stages of blueberry ‘FLS03’ used in this study were collected in Guyue mountain farm, Dongfu Town, Jimei District, Xiamen City, Fujian Province, China. Blueberries at green, pink, red, purple, and blue developmental stages were collected according to the standard of Sun et al. [57]. Collected samples were taken back to the laboratory, surface cleaned with deionized water, pre-cooled in liquid nitrogen, and stored in a −80 °C refrigerator for RNA isolation.

### 4.2. Identification of Anthocyanin Biosynthesis-Related Blueberry bHLH Genes

The blueberry gDNA, cDNA, and protein sequences were downloaded from the blueberry genome website (https://www.vaccinium.org/analysis/49 (accessed on 1 June 2020)). In addition, the recently published anthocyanin biosynthesis-related bHLH protein sequences were downloaded from NCBI (https://www.ncbi.nlm.nih.gov/ (accessed on 20 June 2020)), and were used for local blast searches against the blueberry protein data by using the Blastp program under the criterium of 0.00001. Sequences with an e-value of 0 or the highest bit score were selected as candidate blueberry anthocyanin biosynthesis-related bHLH members. After the bHLH-MYC_N domain (PF14215.5) verification using CDD, the retained blueberry bHLHs were named according to their homologous proteins.

### 4.3. Bioinformatic Analysis of bHLH Genes and Their Encoded Proteins

The online software EXPASY (https://web.expasy.org/protparam/ (accessed on 24 June 2020)), SignalP (http://www.cbs.dtu.dk/services/SignalP-4.0/ (accessed on 24 June 2020)), TMHMM Server 2.0 (http://www.cbs.dtu.dk/services/TMHMM/ (accessed on 24 June 2020)) and WoLFPSORT (https://wolfpsort.hgc.jp/ (accessed on 24 June 2020)) were used for the physiochemical property, signal peptide, transmembrane structure and subcellular localization prediction analysis of blueberry bHLH proteins, respectively [40]. The global sequence alignment procedure Needle embedded in EMBOSS (https://www.ebi.ac.uk/Tools/psa/emboss_needle/ (accessed on 24 June 2020)) was applied for the protein sequence alignment and similarity comparison analysis of blueberry bHLHs. GSDS (http://gsds.cbi.pku.edu.cn/ (accessed on 24 June 2020)) was used to show the gene structures of blueberry *bHLHs* gDNAs. MEME (https://meme-suite.org/meme/tools/meme (accessed on 2 October 2020)) was applied for the conserved motif identification using parameters set as follows: the minimum length = 6, the maximum length = 100, and e-value < 0.00001. The Amazing Simple SeqLogo program embedded in TBtools was used to draw logos for the conserved bHLH domain [58]. The Muscle program embedded in MEGA 6.06 was used to perform multiple sequence alignment analysis of anthocyanin-related bHLH proteins, and a neighbor-joining phylogenetic tree was constructed using MEGA 6.06 under Poisson model, complete deletion, and Bootstrap = 1000 parameters. According to their gene location information, the *bHLHs* chromosomal locations were visualized using the Gene Location Visualize (Advanced) program in TBtools [58]. Based on the Arabidopsis protein database, possible protein–protein interaction analysis of blueberry anthocyanin biosynthesis-related bHLHs were predicted using STRING (https://string-db.org/ (accessed on 2 October 2020)) with the interaction score set as high confidence (0.700).

### 4.4. Identification and Analysis of cis-Acting Elements and Transcription Factor Binding Sites in Promoter of Blueberry bHLH Genes

The 2000 bp sequences upstream of the blueberry *bHLHs* start codon were extracted from the blueberry genome database and used as promoter sequences. PlantCARE (http://bioinformatics.psb.ugent.be/webtools/plantcare/html/ (accessed on 2 October 2020)) and PlantTFDB (http://planttfdb.cbi.pku.edu.cn/ (accessed on 2 October 2020)) were used to investigate the existence and distribution of *cis*-acting elements and transcription factor binding sites on each promoter, respectively.

### 4.5. Gene Cloning and Sequencing of Blueberry bHLH Genes

Total RNA of all the blueberry samples was isolated using Trizol RNA Extraction Kit (TaKaRa). High quality RNA from all samples was equal-weight mixed and used as template for cDNA synthesis using RevertAid First-strand cDNA synthesis Kit (Thermo Scientific). As *bHLHs* related to anthocyanin synthesis showed very high sequence similarity [59], in this study, primers for only *VcAN1*, *VcbHLH1-1* and *VcbHLH1-2* genes were successfully designed according to their cDNA sequences in the blueberry genome (Table 3). The 25 μL PCR system consisted of 1 μL cDNA, 1 μL each forward and reverse primers, 12.5 μL 2 × Green mix and 9.5 μL ddH_2_O. PCR conditions were as follows: pre-denaturation at 95 °C for 3 min; denaturation at 95 °C for 30 s, annealing at 60–60.5 °C for 30 s, extension at 72 °C for 2.5 min, 35 cycles; final extension at 72 °C for 8 min. PCR products were gel extracted, ligated to pMD18-T vector and transformed into component *Escherichia coli* DH5a cells. Positive clones were selected and sent to Beijing Liuhe Huada Gene Technology Co., Ltd. (Beijing, China) for sequencing verification.

### 4.6. Quantitative Real Time PCR Analysis of Blueberry bHLH Genes

TransScript All-in-One First-Strand cDNA Synthesis SuperMix for qPCR (One-Step gDNA Removal) kit was used to separately synthesize cDNA of different samples. Gene specific primers for *VcAN1*, *VcbHLH1-1* and *VcbHLH1-2* genes used for qRT-PCR were designed according to our sequencing results (Table 3). qRT-PCR experiment was performed on a Bio-Rad CFX96^TM^ real-time quantitative fluorescent PCR instrument. Three biological replicates and three technical replicates were made for each gene. qRT-PCR conditions were set as follows: pre-denaturation at 95 °C for 2 min; denaturation at 95 °C for 10 s, annealing at 59 °C for 20 s, extension at 72 °C for 20 s, 45 cycles. qRT-PCR reaction system consisted of 10 μL SYBR Premix ExTaq^TM^ (TaKaRa) fluorescent dye, 7.4 μL ddH_2_O, 0.8 μL of each upstream and downstream primers, and 1 μL cDNA template. Their relative expression levels in different samples were calculated using the 2^−ΔΔCt^ method using *GAPDH* (Genbank ID: AY123769) as the internal reference gene [60]. Excel was used to calculate the relative expression levels of each gene in different samples. SPSS software was used to analyze the significance of the differences among the genes’ relative expression levels in different samples at the 1% level, and to analyze the correlation coefficient between their relative expression levels and the contents of total phenols, flavonoids and anthocyanins in blueberry fruits [57].

## 5. Conclusions

In this study, six *bHLH* genes related to anthocyanin synthesis were identified from the blueberry genome. Their encoded proteins can be further divided into two groups with significant differences in nucleotide and protein sequences, promoter *cis*-acting elements and TFBS types and numbers, and gene expression patterns. Of note, qRT-PCR analysis revealed that *VcAN1* plays positive roles in anthocyanin biosynthesis regulation not only in fruit, but also in podetium and leaf. Therefore, the exploration of its regulatory roles in blueberry anthocyanin biosynthesis is of great significance. Our study can lay a foundation for the blueberry anthocyanin biosynthesis-related bHLHs in the future, and provide a basis for high anthocyanin blueberry breeding.

## Figures and Tables

**Table 1:** Information for the identified blueberry anthocyanin biosynthesis-related basic helix-loop-helix proteins (bHLHs).

Gene Name	Gene ID	Homologous Gene Name	Homologous Gene ID	Identity/%	Reference
*VcAN1*	VaccDscaff11-processed-gene-379.7	*SmelAN1*	SMEL_009g326640.1.01	77.12	[33]
		*AcbHLH42*	MH643775	74.42	[34]
		*MdbHLH3*	ADL36597.1	65.08	[14,35]
		*Noemi*	Cs5g31400	63.08	[15,16]
		*SlAN1*	KR076778	52.03	[37]
		*IbbHLH2*	itf14g18730.t2	46.45	[11]
*VcbHLH42-1*	VaccDscaff24-augustus-gene-24.28	*AcbHLH42*	MH643775	79.10	[34]
		*AtTT8*	At4G09820	73.21	[13]
		*MdbHLH3*	ADL36597.1	69.03	[14,35]
		*Noemi*	Cs5g31400	67.25	[15,16]
		*BjTT8*	BjuB004115	58.48	[38]
		*SlAN1*	KR076778	54.77	[37]
		*SmelAN1*	SMEL_009g326640.1.01	50.14	[33]
*VcbHLH42-2*	VaccDscaff15-augustus-gene-371.25	*AcbHLH42*	MH643775	79.37	[34]
		*MdbHLH3*	ADL36597.1	69.30	[14,35]
		*Noemi*	Cs5g31400	67.11	[15,16]
		*SlAN1*	KR076778	54.77	[37]
		*SmelAN1*	SMEL_009g326640.1.01	49.72	[33]
*VcbHLH42-3*	VaccDscaff19-augustus-gene-381.30	*AcbHLH42*	MH643775	79.23	[34]
		*MdbHLH3*	ADL36597.1	69.30	[14,35]
		*Noemi*	Cs5g31400	67.11	[15,16]
		*SlAN1*	KR076778	54.77	[36]
		*SmelAN1*	SMEL_009g326640.1.01	49.72	[33]
*VcbHLH1-1*	VaccDscaff28-augustus-gene-45.27	*PalbHLH1*	PAYT030711.1	57.12	[38]
		*PPLS1*	Seita.7G195400	53.85	[17]
		*SmelJAF13*	SMEL_008g319200.1.01	49.69	[33]
		*AtEGL3*	At1G63650	48.23	[39]
		*AtGL3*	At5G41315	47.77	[39]
		*ThMYC4E*	KX914905	37.54	[18]
*VcbHLH1-2*	VaccDscaff44-augustus-gene-0.19	*PalbHLH1*	PAYT030711.1	55.61	[38]
		*SmelJAF13*	SMEL_008g319200.1.01	48.55	[33]
		*AtEGL3*	At1G63650	46.95	[39]
		*AtGL3*	At5G41315	46.42	[39]

**Table 2:** Basic physicochemical properties of the identified blueberry anthocyanin biosynthesis-related bHLHs. CDS: Coding sequence; PI: Isoelectric point; GRAVY: Grand average of hydropathicity.

Protein Name	Gene ID	CDS Length/bp	Protein Size/aa	Molecular Weight/Da	PI	Instability Index	GRAVY	Subcellular Localization
VcAN1	VaccDscaff11-processed-gene-379.7	2328	775	85,474.08	5.55	67.34	−0.595	Nuclear
VcbHLH42-1	VaccDscaff24-augustus-gene-24.28	2190	729	80,213.15	5.46	69.43	−0.618	Nuclear
VcbHLH42-2	VaccDscaff15-augustus-gene-371.25	2190	729	80,320.22	5.45	70.96	−0.64	Nuclear
VcbHLH42-3	VaccDscaff19-augustus-gene-381.30	2190	729	80,280.2	5.49	69.96	−0.629	Nuclear
VcbHLH1-1	VaccDscaff28-augustus-gene-45.27	1803	600	67,581.48	6.25	55.99	−0.388	Nuclear
VcbHLH1-2	VaccDscaff44-augustus-gene-0.19	1860	619	69,831.17	6.16	56.26	−0.358	Nuclear

**Table 3:** Information for the primers used in this study.

Target Gene	Primer Name	Primer Sequence	Target Length (bp)	Tm (°C)	Application
*VcAN1*	VcAN1-F	GCAACCCTCTCTCTTTCACT	2885	60.5	Gene cloning
	VcAN1-R	CCATTTCATTACCGCAAGC			
*VcbHLH1-1*	VcbHLH1-1-F	GCACAGAATCAATGGCTTC	2043	60	
	VcbHLH1-1-R	CGTGTTTGTTGGTTTGGC			
*VcbHLH1-2*	VcbHLH1-2-F	TTCCCTTACCCATCTTCCT	2415	60	
	VcbHLH1-2-R	CTGTGTTTGTTGGTTTGGC			
*VcAN1*	VcAN1-qF	CACCCTCCACAGCCTCCGAA	242	59	qRT-PCR
	VcAN1-qR	CTATTCCGCCCCGTGTCAGC			
*VcbHLH1-1*	VcbHLH1-1-qF	CTTGGAATGGGGTGATGGGT	210	59	
	VcbHLH1-1-qR	CACTCGGTATCGGTCAGGTC			
*VcbHLH1-2*	VcbHLH1-2-qF	AGCTGGAGAGAAGGGTCGAA	155	59	
	VcbHLH1-2-qR	GTCCCGTGCTTTCCTCTTGT			
*GAPDH*	GAPDH-qF	ACTACCATCCACTCTATCACCG	116	59	Reference gene
	GAPDH-qR	AACACCTTACCAACAGCCTTG			

## Data Availability

The data presented in this study are available on request from the corresponding authors.
